# Medicinally Used Asarum Species: High-Resolution LC-MS Analysis of Aristolochic Acid Analogs and *In vitro* Toxicity Screening in HK-2 Cells

**DOI:** 10.3389/fphar.2017.00215

**Published:** 2017-05-22

**Authors:** Johanna Michl, Olusheyi Bello, Geoffrey C. Kite, Monique S. J. Simmonds, Michael Heinrich

**Affiliations:** ^1^Research Cluster Biodiversity and Medicines, UCL School of PharmacyLondon, UK; ^2^Royal Botanic GardensRichmond, UK

**Keywords:** metabolomics, LC-MS, nephrotoxicity, *Asarum*, aristolactam

## Abstract

Species of *Asarum* are used in traditional Chinese medicine and, similar to members of the genus *Aristolochia*, they contain aristolochic acid analogs (AAAs). These compounds are known for their nephrotoxic and carcinogenic effects. So far, the phytochemistry and nephrotoxicity of species of *Asarum* is not well studied. A high-resolution LC-MS-based metabolomic approach was used to study the phytochemical variation in medicinally used *Asarum* species. The cytotoxicity of the samples was assessed using human kidney (HK-2) cells. The majority of samples contained potentially nephrotoxic AAAs, including 9-methoxy aristolactam (AL) IV, AL I, and AL IV. These compounds were present in methanol as well as water extracts. AAAs were detected in all parts of the plant. The majority of the extracts were not cytotoxic to HK-2 cells at the doses tested. However, other mechanisms relating to aristolochic acid nephropathy and cancer development, such as DNA adduct formation may occur. The results of this study provide a model for assessing lesser-known plant species for toxicity.

## Introduction

Species of the genus *Asarum* are used as herbal medicines in many parts of the world, including Europe and Asia. The Chinese Pharmacopeia lists the roots and rhizomes of *Asarum heteropoides* f. *mandshuricum* (Maxim.) Kitag, and *Asarum sieboldii* Miq. under the Pin Yin name Xixin (Achenbach and Fischer, 1997). In Europe *Asarum europaeum* L. is used in homeopathic tinctures (Nitzsche et al., 2013) and in Canada and the USA *Asarum canadense* L. was used by Native Americans (Moermon, 2017).

Like the related genus *Aristolochia* (which is also listed in the Chinese Pharmacopeia), *Asarum* contain aristolochic acids and aristolactams (Mix et al., 1982; Kumar et al., 2003). These nitrophenanthrene derivates have nephrotoxic and carcinogenic effects (Michl et al., 2014). Species of *Aristolochia* have become a key concern in healthcare as they are associated with aristolochic acid nephropathy (AAN), a renal fibrosis often associated with upper urothelial cancer (UUC; Chen et al., 2012). It is estimated that in China alone 100 million people may be at risk of developing AAN (Hu et al., 2004; Grollman, 2013).

Species of *Asarum* are generally considered to be less toxic than species of *Aristolochia*. However, a few cases of *Asarum*-related AAN have been reported. In one case report a male patient displayed subacute renal failure after ingesting a herbal powder containing Xixin (Yang et al., 2006). A case of acute poisoning due to the intake of *A. europaeum* has been reported in Switzerland (Jaspersen-Schib et al., 1996). Surprisingly, only eight cases of *Asarum*-related AAN have been reported in the last 45 years (Kim et al., 2013). Like *Aristolochia*-related AAN, it is likely that health practitioners failed to identify the link between nephropathy or tumor development and the exposure to these plants.

Aristolochic acid I (AA I) and aristolochic acid II (AA II) are considered to be the cause of these nephrotoxic and carcinogenic effects (Nortier et al., 2000; Balachandran et al., 2005; Jelakovic et al., 2012). After reductive metabolic activation into aristolactams (ALs), AA I and AA II form DNA adducts, which were found in renal tissues of patients. A large number of *in vitro* and *in vivo* studies showed that AA I and AA II are toxic (Mengs, 1988; Arlt et al., 2011; Yang et al., 2011; Michl et al., 2014). However, they are not necessarily the only (or most potent) toxins present in *Aristolochia* and related genera (Michl et al., 2016). At least 178 aristolochic acid analogs (AAAs) exist, many of which are aristolactams. It is unclear whether these compounds are also able to form DNA adducts. Their possible implications in AAN may have been overlooked (Michl et al., 2014). Apart from AA I and AA II, other compounds may contribute to processes that lead to renal damage (Li et al., 2004; Wen et al., 2006) and carcinogenesis.

Species of *Asarum* generally contain lower amounts of AA I and AA II than *Aristolochia* species (Hashimoto et al., 1999; Chan et al., 2003, 2006; Yuan et al., 2007). Yet, high amounts of AA I (3376.9 ng/mg) were reported in *Asarum crispulatum* C.Y. Cheng and C.S. Yang (Jong et al., 2003). According to Zhao et al. (2008) aerial parts of Xixin herbs contained higher levels of AA I than the roots. Methanol extracts typically contained more AA I than water extracts. A second study by Hsu et al. (2009) found that the amounts of AA I in leaves were the highest followed by petioles, rhizomes and roots.

While a number of studies assessed the amounts of AA I and AA II in *Asarum* spp., little is known about the effects of the entire (small molecule) metabolome and specifically other AAAs. For example, although other compounds, such as AL I are often found in higher amounts in *Asarum* than in *Aristolochia* (Yuan et al., 2008), the Chinese Pharmacopoeia still lists roots and rhizomes of *Asarum* for medicinal use. Furthermore, only the decoction of the root portion is recommended for usage. However, it is questionable as to whether current recommendations for the medicinal uses of *Asarum* species are justified.

The aim of this work is to assess the metabolomic profile and *in vitro* toxicity of medicinally used species of *Asarum* and to evaluate whether current recommendations for their use are appropriate. Therefore, we utilized a systems biology approach to establish the full range of AAAs in a series of *Asarum* species. We carried out a LC-MS-based metabolomic study to compare the secondary metabolites of *Asarum* samples originating from different species, different plant parts, as well as obtained through different extraction techniques. We assessed the cytotoxicity of these extracts in HK-2 kidney cells and studied the relationship between the plants' metabolic profiles and their *in vitro* toxicity using statistical approaches. In a wider context, the current work can be used as a model for assessing toxicity of medicinal plant species, and for elucidating bioactive principles of medicinal plants.

## Materials and methods

### Plant material

*Asarum* samples were mainly obtained from commercial sources in the UK, China, Taiwan or Austria (Table 1). Additional samples were obtained from the living collection at the Royal Botanic Gardens (RBG), Kew, UK and at the botanical garden at Dresden University of Technology (DUoT), Dresden, Germany. Further plant material from the Economic Botany Collection and the Chinese Medicinal Plants Authentication Centre (CMPAC) at RBG, Kew, UK was included in the analysis. When possible, plant material was identified to the species level. However, if no flowering parts were present in the sample, the species name under which the sample was traded is listed in brackets in Table 1. Fresh plant material from RBG Kew and DUoT was freeze-dried and stored at room temperature until further analysis. All other samples were stored at room temperature.

**Table 1:** **Information about *Asarum* samples**.

**No**.	**Accession No**.	Species^*^	**Plant part**	**Origin**
1	22795	Xixin (*A*. sp.*)*	Root/rhizome	Austria^+^
2	22796	Xixin *(A*. sp.*)*	Root/rhizome	Austria^+^
3	22797	Xixin *(A*. sp.*)*	Root/rhizome	Austria^+^
4	22798	Xixin *(A*. sp.*)*	Leaf	Austria^+^
5	22799	Xixin extract *(A*. sp.*)*	Root/rhizome water extract	Austria^+^
6	22800	Xixin *(A*. sp., possibly *A. sieboldii* Miq.)	Root/rhizome	China^+^
7	22800	Xixin (*A*. sp., possibly *A. sieboldii* Miq.)	Leaf	China^+^
8	22801	Xixin (*A*. sp., possibly *A. heterotropoides* Fr. Schmidt. Var. *Mandshuricum* (Maxim.) Kitag)	Root/rhizome	China^+^
9	22802	Xixin (*A*. sp., possibly *A. sieboldii* Miq.)	Root/rhizome	China^+^
10	22803	Xixin (*A*. sp., possibly *A. heterotropoides* Fr. Schmidt. Var. *mandshuricum* (Maxim.) Kitag.)	Root/rhizome	China^+^
11	22805 (1984–4852)	*A. caulescens* Maxim.	Leaf	LC Kew
12	22806 (1969–18007)	*A. europaeum* L.	Leaf	LC Kew
13	22807 (1978–2742)	*A. caudatum* Lindl.	Leaf	LC Kew
14	22808 (170–3809)	*A. canadense* L.	Leaf	LC Kew
15	22809 (2000–2631)	*A. splendens* (F.Maek.) C. Y. Chen and C. S. Yang	Leaf	LC Kew
16	22810	Xixin extract (*A*. sp.)	Root/rhizome water extract	UK^+^
17	22811	Xixin extract (*A*. sp.)	Root/rhizome water extract	UK^+^
18	22812	Xixin (*A*. sp.)	Root/rhizome	UK^+^
19	22825	Xixin (*A*. sp.)	root/rhizome	UK^+^
20	22825	Xixin (*A*. sp.)	Leaf	UK^+^
21	22813	Xixin (*A*. sp.)	Stem	UK^+^
22	22813	Xixin (*A*. sp.)	Leaf	UK^+^
23	22814	Xixin (*A*. sp.)	Root/rhizome	UK^+^
24	22815	Xixin (*A*. sp.)	Root/rhizome	UK^+^
25	22815	Xixin (*A*. sp.)	Leaf	UK^+^
26	22816	*A. sagittarioides* C. F. Liang	Leaf	Austria
27	20327	Xixin (*A*. sp., *possibly A. sieboldii* Miq. Var. *seolense* Nakai)	Root/rhizome	Taiwan^+^
28	20326	Xixin (*A*. sp., possibly *A. heterotropoides* Fr. Schmidt var. *mandshuricum* (Maxim.) Kitag.)	Root/rhizome	Taiwan^+^
29	22817 (EBC 47625)	Xixin (*A*. sp., possibly *A. sieboldii* Miq.)	Root/rhizome	EBC Kew^+^
30	22818 (EBC 45660)	Xixin (*A*. sp., possibly *A. sieboldii* Miq.)	Root/rhizome	EBC Kew^+^
31	22819 (EBC 47604)	Xixin (*A*. sp., possibly *A. sieboldii* Miq.)	Root/rhizome	EBC Kew^+^
32	22820 (EBC 80573)	*A. heterotropoides* Fr. Schmidt var. *mandshuricum* (Maxim.) Kitag.	Root/rhizome	EBC Kew^+^
33	22820 (EBC 80574)	*A. heterotropoides* Fr. Schmidt var. *mandshuricum* (Maxim.) Kitag.	Root/rhizome	EBC Kew^+^
34	22821 (EBC 81420)	Xixin (*A*. sp.)	Root/rhizome	EBC Kew^+^
35	22822 (EBC 81093)	*A. sieboldii* Miq.	Root/rhizome	EBC Kew^+^
36	22823 (EBC 81424)	*A. sieboldii* Miq.	Leaf	EBC Kew^+^
37	22824 (EBC 81480)	Xixin (*A*. sp.)	Leaf	EBC Kew^+^
38	20795	*A. cardiophyllum* Franch.	Leaf	DUoT

*Samples were obtained as commercial products in China, Taiwan, the UK or Austria, through the Economic Botany Collection (EBC), Royal Botanic Gardens (RBG), Kew, the living collection (LC), Royal Botanic Gardens, Kew or the LC at Dresden University of Technology (DUoT)*.

* For non-authenticated species, the likely taxon is given in brackets.

+ *Commercial samples*.

### Chemicals

The chemicals used for the LC-DAD-ESI-MS analysis were methanol, acetonitrile, formic acid (all obtained from Thermo Fisher Scientific, MA, USA). Reference standards for AA I and AA II were isolated from *Aristolochia repens* Mill. AA C was purchased from Phytolab (Germany). AL I was isolated from *A. sieboldii* Miq. The purities of all reference standards were determined using HPLC.

### Extraction of the plant material

The dried plant material was ground in a mortar and for each sample 50 mg of dried plant material was extracted with 1 mL of 70% aqueous methanol in an 1.5 mL Eppendorf microtube. After about 14 h extraction at room temperature the extracts were centrifuged at 6,600 relative centrifugal force (RCF) for 10 min. The supernatant liquid was filtered using Whatman 0.45 μm polytetrafluoroethylene (PTFE) filters and 700 μL of the sample solution were used for LC-DAD-ESI-MS measurements. The samples 3, 8, and 35 were extracted and analyzed in triplicate to demonstrate repeatability of the extraction method.

### LC-DAD-ESI-MS analysis

The analysis was carried out using a Thermo Scientific HRAM LC-MS system (Thermo Scientific, MA, USA) consisting of an “Accela” liquid chromatograph with diode array detector and an “LTQ-Orbitrap XL” hybrid linear ion trap-orbitrap mass spectrometer. Chromatography was performed on a 150 × 3 mm, 3 μm Phenomenex Luna C18 column using a 400 μL/min flow rate. The separation was obtained using the following linear mobile phase gradient: 0 min: 90% A, 0% B, 10% C; 50 min: 0% A, 90% B, 10% C, 55 min: 0% A, 90% B, 10% C, 57 min: 90% A, 0% B, 10% C, 60 min 90% A, 0% B, 10% C (A: water, B: methanol, C: acetontirile containing 1% formic acid). The injection volume was 5 μL. Ammonia solution was added post column at a flow rate of 0.1 μL/min. The mass spectrometer was fitted with an electrospray source (Thermo “Ion Max”) operated in positive mode at a source voltage of 3.5 kV using sheath and auxiliary nitrogen flow rates of 60 and 20 units, respectively, and a capillary temperature of 300°C. High resolution MS1 scanning was performed in the orbitrap (*m/z* 250–2000; resolution 30,000). Low resolution data dependent MS2 scans were undertaken in the linear ion trap (isolation width 4 *m/z* units; collision energy 35%).

### Data reduction and multivariate data analysis

Filtering, peak extraction, chromatogram de-convolution and peak alignment of the high-resolution LC-MS data were performed automatically using Mzmine 2 (http://mzmine.sourceforge.net). Peaks between 0 and 50 min retention time with a minimum peak intensity of 50,000 and *m/z* between 200 and 650 were extracted. Only the 1,000 metabolites with the highest average peak areas across all samples were used for further data analysis. This process resulted in a data set consisting of 38 observations (samples) and 1000 variables (metabolites). Before PCA, the resulting data set was normalized to the total raw signal. PCA and OPLS-DA was carried out on the normalized and pareto scaled dataset using the software SIMCA P+ (v. 12, Umetrics, Umea, Sweden).

### Metabolite identification assignment

For the peaks extracted by Mzmine 2, the ion species (i.e., adducts) were determined so as to obtain an experimental accurate molecular mass. This was then searched against the CRC Dictionary of Natural Products to identify candidate compounds having molecular masses within 5 ppm of the experimental value. AA I, AA II, AA III, AA IIIa, AA IV, AA D, AA IIIa-6-β-d-glucoside, aristolochin, and AL I were identified by comparison to reference standards. Further, compounds were identified by comparing accurate mass data to an in-house database (UCL School of Pharmacy, London, UK) consisting of 178 AAAs, which have previously been isolated from natural sources. Putative assignments for those AAAs were made based on their accurate mass, retention time, mass fragmentation, and UV spectra.

### Cell culture conditions

HK-2 cells (American Type Culture Collection, Manassas, VA, USA) were maintained in Dulbecco's modified eagle medium (DMEM, Gibco Invitrogen) supplemented with 10% fetal bovine serum (FBS, Gibco Invitrogen), 100 U/mL penicillin and 100 μg/mL streptomycin (Gibco Invitrogen). All cells were cultured in a humidified atmosphere with 5% CO_2_ at 37°C. For routine cultivation, cells were maintained up to 80% of confluency and the medium was replaced every 3 days.

### Sulphorhodamine B (SRB) assay

Cytotoxicity assessment of the plant extracts was carried out using the SRB assay (Houghton et al., 2007). HK-2 cells (10^4^ cells/well) were plated in 96-well plates overnight. Afterwards the medium was replaced by medium containing the plant extracts dissolved in dimethyl sulfoxide (DMSO, VWR, Poole, UK). The cells were treated with plant extracts for 72 h at concentrations of 0, 12.5, 25, 50 100, and 200 μg/mL. The final concentration of DMSO in the medium did not exceed 1% v/v. Afterwards the cells were fixed with 40% trichloroacetic acid (TCA, Sigma, St. Louis, MO, USA) for at least 1 h. The fixed cells were washed with deionized water and stained with 0.4% SRB (Sigma, St. Louis, MO, USA) in 1% acetic acid (Fisher Scientific, Loughborough, UK) for 30 min. Afterwards the cells were washed with 1% acetic acid to remove excess color. Plates were dried and then dissolved in 30 mM Tris base (Sigma, St. Louis, MO, USA). Cell growth was quantitated based on the amount of SRB measured by a spectrophotometer (Tecan Infinite M200 plate reader, Tecan, Maennedorf, Switzerland) at 490 nm. Three independent experiments were carried out, each carried out in technical triplicates. IC_50_ values were obtained using the curve fit through non-linear regression function in GraphPad Prism 6.0 (GraphPad Software, San Diego, CA, USA). Colchicine (10 μg/mL, Sigma, St. Louis, MO, USA) was used as a positive control to ensure the performance of the assay.

## Results

### Phytochemical variation in Xixin samples using non-targeted LD-DAD-ESI-MS

We obtained chromatographic information from the non-targeted metabolomic analysis of 38 *Asarum* samples (Table 1) in positive-ionization mode of high resolution accurate mass LC-MS (HRAM LC-MS). We preprocessed the information to extract chromatographic features. Matching features from different samples were aligned. Normalization and pareto scaling resulted in the final data set for subsequent statistical analysis.

**Figure 1:**
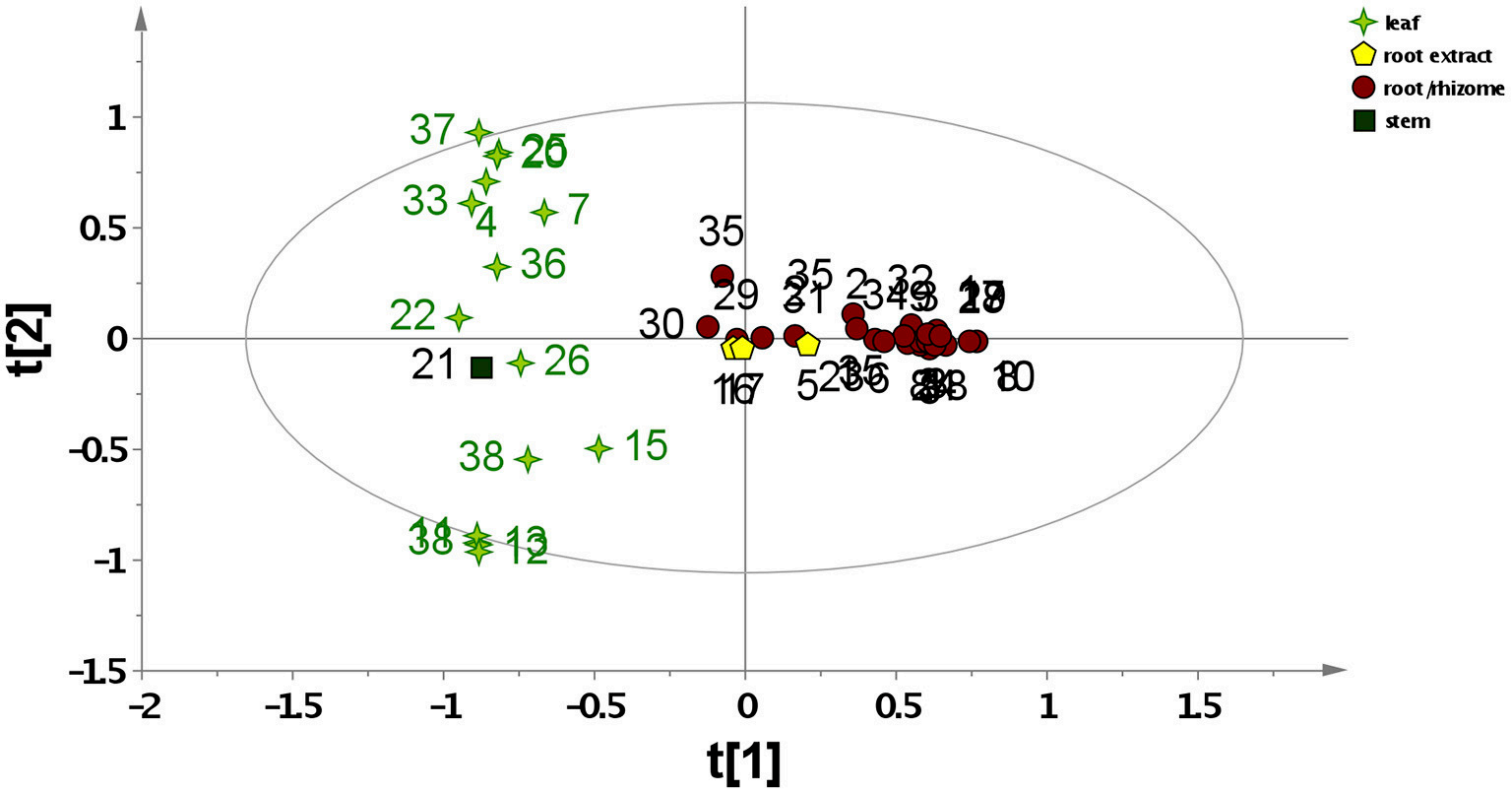
**PCA scores plots based on LC-MS data for all *Asarum* samples**. The samples are labeled according to their sample number (Table 1) and different symbols were assigned for different plant parts or extraction methods (data normalized to the total raw signal, mean-centered, and pareto scaled).

To study the variability within the data set, principal component analysis (PCA) was carried out. A 2-component PCA model explained 23.6% of the total variance. The PCA scores plot in Figure 1 shows a clear separation between *Asarum* leaf and root material. However, different modes of preparation (methanolic vs. water extracts) were not differentiated. All leaf samples, as well as sample 21 [Xixin, *A*. sp. (stem)] were clearly separated (left hand side of the plot) from the root samples. This included the commercial root extracts, which are generally extracted using water decoction (right hand side of the plot). Thus, the metabolic profile of *Asarum* extracts is largely dependent on the part of the plant used, rather than genetic variability (species) or extraction method. Samples 11–14 originate from leaves of different species of *Asarum* (*A. caulescens, A. europaeum, A. caudatum*, and *A. canadense*) and have similar metabolite profiles. Since many samples used in traditional Chinese medicine are only available in the form of botanical drugs, these were not identified to species level (Table 1). However, since most of these samples were traded under the Pin Yin name Xixin, it is likely that they originate from either *A. sieboldii* or *A. heterotropoides*. From the PCA scores plot, it is evident that the phytochemistries of *A. sieboldii* and *A. heterotropoides* are similar (Figure 1). For example, samples 33 and 35 (*A. heterotropoides* (root) and *A. sieboldii* (root), respectively) are located in close proximity in the scores plot.

The PCA loadings plots were used to identify metabolites responsible for the differentiation in the PCA analysis (Figure S1). Samples with positive PC1 scores (such as the majority of root/rhizome samples) are characterized by a major metabolite giving *m*/*z* at 248.2012 (*t*_R_ = 40.63 min), assigned as a protonated molecule from the presence of a confirmatory sodiated molecule at *m/z* 270.1831, both of which suggest a molecular formula of C_16_H_25_NO (e.g., calc. for C_16_H_26_NO^+^ = *m/z* 248.2009; 1.2 ppm error). A possible candidate is a mixture of (2E,4E,8Z,10E)-*N*-isobutyl-2,4,8,10-dodecatetraenamide and (2E,4E,8Z,10Z)-*N*-isobutyl-2,4,8,10-dodecatetraenamide, which was previously isolated from *A. sieboldii* (Quang et al., 2012; Wen et al., 2014), *Asarum heterotropoides* (Quang et al., 2012) and *Asarum forbesii* Maxim (Zhang et al., 2005). These candidates are supported by major fragments in the MS/MS spectrum of the protonated molecule at *m*/*z* 175.119 (C_12_H_15_O^+^), 149.1326 (C_11_${\text{H}}_{17}^{+}$) and 142.1227 (C_8_H_16_NO^+^), which can be rationalized against the structures. Samples with negative PC1 scores (such as all leaf samples) contain higher amounts of a compound giving *m*/*z* at 277.2164 (*t*_R_ = 42.76 min). This signal can be assigned to the [M+H]^+^ ion from a confirmatory [M+NH_4_]^+^ ion at *m/z* 294.2430, both suggesting a molecular formula of C_18_H_28_O_2_ (e.g., calc for C_18_H_29_${\text{O}}_{2}^{+}e\;=$ 277.2162; 0.6 ppm error). Possible candidates for this compound are various octadecatetraenoic acids. The differentiation of the *Asarum* samples by PC2 is more complex (Figure S2). Negative PC2 scores correspond to a metabolite giving a protonated molecule at *m*/*z* 597.1815 (*t*_R_ = 16.45 min). This is likely to be 2′,4,4′,6′-tetrahydroxychalcone-2′,4′-di-*O*-β-D-glucopyranoside (chalcononaringenin 2′,4′-di-*O*-glucoside), a flavonoid previously isolated from *Asarum canadense* L. (Iwashina and Kitajima, 2000). This identification was supported by a match of the MS3 (*m*/*z* 597 → 273) spectrum with the MS2 (*m*/*z* 273) spectrum of chalcononaringenin an in-house library. Further, supporting this identification is the failure of the sodiated molecule to lose an aglycone moiety following MS/MS (suggesting the sugars were substituted at two positions). In contrast, positive PC2 scores correspond to *m*/*z* 277.2164 (*t*_R_ = 42.76 min; an octadecatetraenoic acid as discussed above) and *m*/*z* 295.2269 (*t*_R_ = 44.21 min; the protonated molecule of a hydroxyoctadecatrienoic acid).

### Variation in AAAs

**Figure 2:**
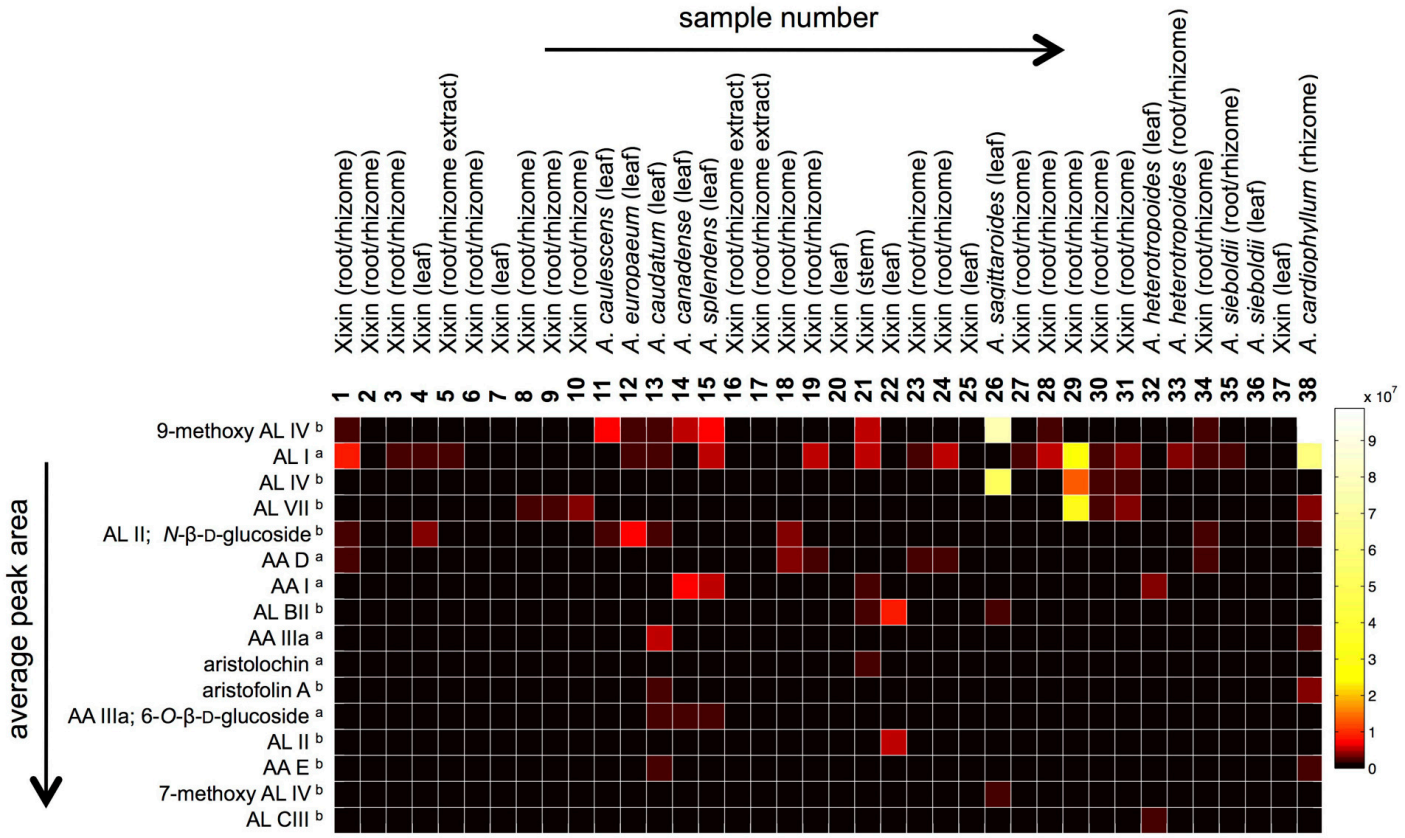
**Heat map comparing relative LC-MS peak areas of identified aristolochic acid analogs**. Information about the origin of the samples is given in Table 1. The compounds (rows) are sorted by descending average peak areas across all samples (^a^identified by comparison with reference standard; ^b^Tentative assignment based on accurate mass, UV spectra and mass fragmentation).

In order to study the variation in AAAs content, targeted metabolite profiling was carried out. Several AAAs (AA I, AL I, AA IIIa, AA D, AA IIIa 6-*O*-β-d-glucoside, and aristolochin) could be identified by comparison to reference standards. However, tentative assignments based on accurate mass, retention times, UV maxima, and fragmentation ions obtained through LC-DAD-ESI-MS analysis were made for other AAAs (Table 2, Figure 2). Table 2 shows the 16 most commonly occurring AAAs, which could be identified within the set of analyzed samples. 9-Methoxy AA IV (or one of its isomers) was found in the highest average amount across all samples. However, while this compound was found in large amounts in species such as *A. cardiophyllum*, and *A. saggitaroides*, it was detected in only a few of the commercial Xixin samples (*A*. sp. *A. sieboldii* and *A. heterotropoides*). Within the analyzed Xixin samples, AL I was the most common aristolochic acid analog. However, a variety of other aristolactams such as AL IV and its isomer AL VII, as well as AL II and *N*-β-d-glucoside were detected. While AA I was only detected in seven samples (4, 14, 15, 21, 23, 32, and 36), AA II was not detected at all. AA I was found in high amounts in *A. canadense* and *A. splendends*. AA I occurred only as a minor compound within Xixin samples, with the average amount being higher in aerial parts compared to roots; but in contrast to previous reports^22^ these differences were not statistically significant (*p* = 0.11). On the other hand, the amounts of AL I in Xixin root samples were significantly higher, compared to aerial parts (*p* = 0.03). This finding has not previously reported, since AL I was only quantified in a few studies. Although AL I is a rather non-polar compound and therefore poorly soluble in water, it was also detected in water based commercial root decoctions. This suggests that AL I can be extracted into water when heat is used during the extraction process, or it could be mediated through the formation of ion-pairs with other substances in the extracts. Although AA I is more water soluble than ALI, AA I was not detected in any of the three root decoction samples.

**Table 2:** **Retention times, UV maxima and fragmentation ions of identified aristolochic acid analogs**.

**No**.	**Compound**	**Retention time (min)**	**[M+H]^+^ (*m*/*z*)**	**[M+NH_4_]^+^(*m*/*z*)**	**Fragmentation ions (*m/z*)^c^**	**UV maxima (nm)**
1^b^	9-methoxy aristolactam IV (9-methoxy AL IV)	34.61	354.0975		339, 324, 293, 266	236, 271, 287, 343, 393
2	aristolactam I^a^ (AL I)	36.36	294.0759		279, 251	236, 291, 328, 391
3^b^	aristolactam IV (AL IV)	36.46	324.0867		309, 280, 266	235, 263, 335, 406
4^b^	aristolactam VII (AL VII)	34.78	324.0867		309, 294, 266	234, 304, 414
5	aristolactam II; *N*-β-d-glucoside^b^ (AL II; *N*-β-d-glucoside)	25.40	426.1192		264, 234, 206	234, 277, 330, 274
6	aristolochic acid D^a^ (AA D)	30.55		375.0821	357, 312, 297, 267	238, 329, 408
7	aristolochic acid I^a^ (AA I)	36.07		359.0870	342, 324, 298, 296, 281, 268	252, 320, 392
8^b^	aristolactam BII (AL BII)	35.74	280.0972		265, 264, 236, 234	235, 276, 317, 379
9	aristolochic acid IIIa^a^ (AA IIIa)	30.92		345.0713	328, 310, 284, 254	235, 321, 400
10	aristolochin^a^	21.94		537.1352	520, 474, 314, 312	240, 325, 404
11^b^	aristofolin A	24.82		537.1353	474, 358, 312, 297	234, 325, 394
12	aristolochic acid IIIa; 6-*O*-β-d-glucoside^a^ (AA IIIa; 6-*O*-β-d-glucoside)	23.20		507.1252	490, 327, 310, 284, 266	252, 304, 390
13	aristolactam II^b^ (AL II)	34.86	264.0660		234, 206, 179,	235, 277, 325, 374, 392
14	aristolochic acid E^b^ (AA E)	31.94		375.0826	358, 312, 297, 267	237, 326, 408
15^b^	7-methoxy aristolactam IV (7-methoxy AL IV)	35.42	354.0976		324, 293, 295, 266	234, 268, 386
16	aristolactam CIII^b^ (AL CIII)	25.88	340.1181		308, 280	236, 293, 392

*The compounds are sorted by descending average peak areas across all samples*.

a *Identified by comparison with reference standard*.

b *Tentative assignment based on accurate mass, UV spectra, and mass fragmentation*.

c *Fragmentation ions were extracted from MS2 and MS3 scans*.

### *In vitro* toxicity of *Asarum* extracts

The cytotoxicity of the *Asarum* samples in HK-2 cells was assessed after treatment for 72 h using the sulphorhodamine B (SRB) assay (Houghton et al., 2007). Several *Asarum* extracts showed cytotoxic effects in HK-2 cells (Table 3). For example, sample 12 [*A. europaeum* (leaf)] and sample 18 [Xixin, *A*. sp. (root)], exhibited IC_50_ values of 44.10 and 57.01 μg/mL, respectively. This is comparable to the cytotoxicity of species of *Aristolochia*, which was previously assessed in HK-2 cells. However, the majority of the tested samples did not reduce cell viability to fewer than 50% of the solvent control at a concentration of 200 μg/mL.

**Table 3:** **Cytotoxicity (IC_50_ values) after treatment of HK-2 cells with *Asarum* samples for 72 h**.

**Sample No**.	Species^*^	**Cytotoxicity IC_50_[μg/mL]**
1	Xixin (*A*. sp.*)*	>200
2	Xixin *(A*. sp.*)*	>200
3	Xixin *(A*. sp.*)*	>200
4	Xixin *(A*. sp.*)*	>200
5	Xixin extract (*A*. sp.)	>200
6	Xixin *(A*. sp., possibly *A. sieboldii* Miq.)	>200
7	Xixin (*A*. sp., possibly *A. sieboldii* Miq.)	>200
8	Xixin (*A*. sp., possibly *A. heterotropoides* Fr. Schmidt. Var. *Mandshuricum* (Maxim.) Kitag)	>200
9	Xixin (*A*. sp., possibly *A. sieboldii* Miq.)	>200
10	Xixin (*A*. sp., possibly *A. heterotropoides* Fr. Schmidt. Var. *mandshuricum* (Maxim.) Kitag.)	>200
11	*A. caulescens* Maxim.	156.4
12	*A. europaeum* L.	44.1
13	*A. caudatum* Lindl.	140.2
14	*A. canadense* L.	>200
15	*A. splendens* (F.Maek.) C. Y. Chen and C. S. Yang	95.83
16	Xixin extract (*A*. sp.)	>200
17	Xixin extract (*A*. sp.)	>200
18	Xixin (*A*. sp.)	57.01
19	Xixin (*A*. sp.)	83.4
20	Xixin (*A*. sp.)	>200
21	Xixin (*A*. sp.)	>200
22	Xixin (*A*. sp.)	>200
23	Xixin (*A*. sp.)	>200
24	Xixin (*A*. sp.)	77.35
25	Xixin (*A*. sp.)	>200
26	*A. sagittarioides* C. F. Liang	>200
27	Xixin (*A*. sp., *possibly A. sieboldii* Miq. Var. *seolense* Nakai)	135
28	Xixin (*A*. sp., possibly *A. heterotropoides* Fr. Schmidt var. *mandshuricum* (Maxim.) Kitag.)	87.9
29	Xixin (*A*. sp., possibly *A. sieboldii* Miq.)	78.75
30	Xixin (*A*. sp., possibly *A. sieboldii* Miq.)	>200
31	Xixin (*A*. sp., possibly *A. sieboldii* Miq.)	95.85
32	*A. heterotropoides* Fr. Schmidt var. *mandshuricum* (Maxim.) Kitag.	>200
33	*A. heterotropoides* Fr. Schmidt var. *mandshuricum* (Maxim.) Kitag.	>200
34	Xixin (*A*. sp.)	194.5
35	*A. sieboldii* Miq.	>200
36	*A. sieboldii* Miq.	>200
37	Xixin (*A*. sp.)	>200
38	*A. cardiophyllum* Franch.	>200

*Cytotoxicity was assessed using the sulphorhodamine B (SRB) assay*.

* *For non-authenticated species, the likely taxon is given in brackets*.

The AAAs AA I and AA II are considered to be the main compounds responsible for the nephrotoxic effects of species of *Aristolochia* and *Asarum*. In order to investigate, whether there is a link between the amounts of AA I in the samples and their cytotoxicity in HK-2 cells, univariate regression analysis was carried out. However, no correlation was found between AA I peak areas and the extracts IC_50_ values (*R*^2^ = 0.000). This can be explained by the fact that only one of the samples containing AA I [*A. splendends* (leaf)] exhibited cytotoxicity, while no cytotoxicity was observed for all other samples with doses up to 200 μg/mL.

To elucidate the possible bioactive principle behind the cytotoxic effects of the extracts, an untargeted approach was used, where all detected metabolites were taken into account. Therefore, we used orthogonal projection to latent structures (OPLS) regression analysis. In contrast to univariate regression analysis, where the correlation between only one X and one Y variable is assessed, OPLS is a method for relating two data matrices (X and Y) within a linear multivariate model. In this study, the X matrix consisted of 38 observations (samples) and 1,000 variables (the 1,000 metabolites with the highest average LC-MS peak areas across all samples). However, the Y matrix consisted of only one qualitative variable (0 for non-toxic vs. 1 for toxic extracts). In this case, since the Y matrix consists of discrete values, the method is called OPLS-discriminant analysis (OPLS-DA). The OPLS-DA loadings plots were used to identify which metabolites correlate directly or indirectly, significantly or non-significantly with the Y variable (toxic vs. non-toxic). OPLS-DA showed that toxic samples contain higher amounts of metabolites giving *m*/*z* at 274.2167, 276.2325, and 252.2324. The molecular formulas and MS/MS fragmentations of these ions are in accordance with them being the protonated molecules of various alkylamides. Aristolochic acids and ALs were not found to be important discriminators between toxic vs. non-toxic samples.

## Discussion

*Asarum* species have been used as medicinal plants in China and in other parts of the world. However, so far little is known about their content in aristolochic acids and aristolactams or their potential kidney toxicity. A comprehensive metabolomic approach has shown that the majority of *Asarum* samples contain AAAs considered to be nephrotoxic.

In TCM, plants known to contain toxic compounds are often regarded as safer after preparation of the plant material in a specific way, or only those plant parts known not to contain the toxins are used. Furthermore, monographs on species in national pharmacopeias require time to change and often do not take into account recent advances in the knowledge about the chemistry of the species. This could result in misleading recommendations about the use of some species with potential hazards for public health. The plant genus *Asarum* is one example of this, especially since its true level of usage is poorly known (e.g., in China and Southern Europe). In addition to changing guidelines in national pharmacopeias, more awareness needs to be raised about potential health risks associated with the use of herbal medicine. This will in time lead to changes in the traditional practices.

Previous reports have shown that levels of aristolochic acids were low in decoction of roots and rhizomes of Herba Asari (Zhao et al., 2008) thus it was retained in the Chinese Pharmacopoeia. However, in this study, no significant difference in the levels of AA I was detected in aerial parts of *Asarum* spp. compared to root samples. Relative levels of AL I were found to be even higher in root samples compared to aerial parts, and AL I was also detected in root decoctions. Little is known about the toxicity and carcinogenicity of AL I.

Although most Xixin samples included in this study contained potentially nephrotoxic AAAs, some samples did not contain detectable amounts of these compounds. This could indicate that Xixin samples purchased from markets may not originate from *Asarum* sp., and were either misidentified or replaced with other medicinal plants. The lack of detectable AAAs could also be due to natural variation (genetic, seasonal or ecological) and requires further investigation. Most samples were non-cytotoxic to kidney cells *in vitro*. Interestingly, no correlation was found between the amounts of AA I and their toxicity. However, other mechanisms relating to aristolochic acid nephropathy, such as DNA adduct formation may occur and deserve further research.

The present study has important implications on whether and how the genus *Asarum* should be used medicinally in the future. In contrast to previous studies, where only AA I and AA II were taken into account, we detected other AAAs in root and rhizome samples of *Asarum*. Since little is known about the toxicity of these compounds, we cannot conclude that root and rhizome samples are safe to use. The study also demonstrates that systematic assessment of a group of species' metabolomic profile can provide a basis for a broader assessment of associated risks. Not only are there other compounds than aristolochic acids that need to be taken into consideration, but the entire composition of some of the most active extracts needs to be understood. The study thus serves as a model for assessing closely related species used as traditional medicines. More broadly, the strategy presented here can also be used in identifying new drug leads from medicinal plants (and fungi).

## Author contributions

JM, OB, and GK carried out the experiments. JM and OB obtained sample materials. JM and MH wrote the manuscript. JM, MS, and MH designed the study.

## Funding

We thank the Bloomsbury Colleges (University of London) for funding this project.

### Conflict of interest statement

The authors declare that the research was conducted in the absence of any commercial or financial relationships that could be construed as a potential conflict of interest.
