# Supplementary material for: Medicinally Used Asarum Species: High-Resolution LC-MS Analysis of Aristolochic Acid Analogs and In vitro Toxicity Screening in HK-2 Cells
Source: Front Pharmacol. 2017 May 22;8:215. doi: 10.3389/fphar.2017.00215 (PMC5439001; doi:10.3389/fphar.2017.00215)
Supplement: Supplementary file 1 [file DataSheet1.DOCX]

**Supporting Information**

**Medicinally Used *Asarum* Species: Metabolomic Analysis of the Aristolochic Acid Analogues and *in vitro* Nephrotoxicity**

Johanna Michl^1^, Olusheyi Bello^1^, Geoffrey C. Kite^2^, Monique S. J. Simmonds^2^, Michael Heinrich^1,^*

^1^Research Cluster Biodiversity and Medicines, UCL School of Pharmacy, London, United Kingdom

^2^Royal Botanic Gardens, Kew, Richmond, United Kingdom

Figure S1: PC1 loadings plots

Figure S2: PC2 loadings plots
